# South African Children: A Matched Cohort Study of Neurodevelopmental Impairment in Survivors of Invasive Group B *Streptococcus* Disease Aged 5 to 8 Years

**DOI:** 10.1093/cid/ciab814

**Published:** 2021-11-02

**Authors:** Lois M Harden, Shannon Leahy, Sanjay G Lala, Proma Paul, Jaya Chandna, Sarah Lowick, Sibongile Mbatha, Tamara Jaye, Barbara Laughton, Azra Ghoor, Pamela Sithole, Jacqueline Msayi, Ntombifuthi Kumalo, Tshepiso N Msibi, Shabir A Madhi, Joy E Lawn, Ziyaad Dangor

**Affiliations:** 1 Brain Function Research Group, School of Physiology, Faculty of Health Sciences, University of the Witwatersrand, Johannesburg, South Africa; 2 Department of Paediatrics and Child Health, Faculty of Health Sciences, University of the Witwatersrand, Johannesburg, South Africa; 3 Maternal, Adolescent, Reproductive & Child Health (MARCH) Centre, London School of Hygiene & Tropical Medicine, London, UK; 4 Department of Infectious Disease Epidemiology, London School of Hygiene & Tropical Medicine, London, UK; 5 Department of Paediatrics and Child Health, Stellenbosch University, Tygerberg, Western Cape, South Africa; 6 South African Medical Research Council: Vaccines and Infectious Diseases Analytics Research Unit, Faculty of Health Sciences, University of the Witwatersrand, Johannesburg, South Africa

**Keywords:** group B *Streptococcus*, neurodevelopment, neonatal meningitis, neonatal sepsis, Griffiths Mental Development Scales

## Abstract

**Background:**

Invasive group B *Streptococcus* (iGBS) sepsis and meningitis are important causes of child mortality, but studies on neurodevelopmental impairment (NDI) after iGBS are limited. Using Griffiths Mental Development Scales–Extended Revised (GMDS-ER), we described NDI in iGBS survivors and non-iGBS children from South Africa, as part of a 5-country study.

**Methods:**

We identified children aged 5–8 years with a history of iGBS and children with no history of iGBS between October 2019 and January 2021. Children were matched on sex, and birth data (month, year) (matched cohort study). Moderate or Severe NDI was the primary outcome as a composite of GMDS-ER motor, GMDS-ER cognition, hearing, and vision. Secondary outcomes included mild NDI, any emotional-behavioral problems, and GMDS-ER developmental quotients (DQ) calculated by dividing the age equivalent GMDS-ER score by the chronological age.

**Results:**

In total, 160 children (iGBS survivors, 43; non-iGBS, 117) were assessed. Among iGBS survivors 13 (30.2%) had meningitis, and 30 (69.8%) had sepsis. Six (13.9%) iGBS survivors, and 5 (4.3%) non-iGBS children had moderate or severe NDI. Children who survived iGBS were 5.56 (95% confidence interval [CI]: 1.07–28.93; *P* = .041) times more likely to have moderate or severe NDI at 5–8 years than non-iGBS children. Compared to the non-iGBS children, iGBS meningitis survivors had a significantly lower global median DQ (*P* < .05), as well as a lower median DQ for the language GMDS-ER subscale and performance GMDS-ER subscale (*P* < .05).

**Conclusions:**

Children surviving iGBS, particularly meningitis, are more likely to have NDI at 5–8 years compared to non-iGBS children. Further research is required to improve detection and care for at-risk newborns.

**KEY FINDINGS**

1. WHAT IS KNOWN AND WHAT IS NEW?

Globally, there are about 231 000 early-onset and 161 000 late-onset cases of infant iGBS, with 91 000 deaths, annually. Most studies of iGBS adverse sequelae, have focused on mortality, with limited data describing neurodevelopmental impairment (NDI) beyond early childhood. Additionally, few studies compare NDI with a counterfactual group, especially in Sub-Saharan Africa where disease burden is highest.

2. WHAT DID WE DO AND WHAT DID WE FIND?

As part of a 5-country study, and building on earlier work that described NDI at one-year-of-age in a cohort of iGBS affected South African children, we assessed the prevalence of NDI in 43 children surviving to age 5–8 years and in an age and sex-matched non-iGBS comparison cohort of 117 children using Griffiths Mental Development Scales–Extended Revised (GMDS-ER) and other relevant multi-domain evaluations. We found significantly increased rates of NDI, across multiple domains, in iGBS survivors compared to their non-iGBS comparison peers. We also detected higher than expected rates of mild NDI in the cognitive domain in the non-iGBS comparison children.

3. WHAT TO DO NOW IN PROGRAMMES?

In low- and middle-income countries (LMIC) settings, infants with iGBS disease need follow-up to detect NDI early, and more investment is needed to support the increased health care and educational needs for these children and their families.

4. WHAT NEXT IN RESEARCH?

Further studies are required to develop simpler, standardized, culturally appropriate, and openly accessible NDI assessments to identify milder impairment during primary education school years. Implementation research with economic evaluations is necessary to inform scale up of assessments to detect NDI earlier among at-risk newborns.

Sepsis and meningitis due to invasive group B streptococcal (iGBS) disease during early infancy is an important cause of child mortality, especially in low- and middle-income countries (LMIC) [1]. Recent estimates of the worldwide burden of GBS suggested that there are 231 000 early-onset and 161 000 late-onset iGBS infant cases each year, and 46 000 stillbirths and 91 000 young-infant deaths (Gonçalves BP, Procter SR, Paul P et al, manuscript in preparation). Outcome data for iGBS have mostly focused on deaths, with limited studies describing neurodevelopmental impairment (NDI) beyond early childhood [2]. Studies report NDI after iGBS meningitis and not iGBS sepsis, to a median age of 18 months, and focus on moderate or severe impairment [2].

Children with NDI require more care and educational input, thereby placing greater demands on the caregiver and family. Children also suffer from complications directly attributable to NDI, such as epilepsy or pneumonia, with more frequent hospitalizations and healthcare needs. These demands may be heavier on families in LMIC, given less resources from governments for support. In Soweto, South Africa, there is a dual burden of human immunodeficiency virus (HIV) infection and HIV exposure in young infants and GBS disease (incidence reported as 2.59 per 1000 live births) [3]. The dual exposure of HIV and GBS disease may pose a synergistic risk for NDI burden in South African children [2, 4].

We have previously described abnormal neurological development in 15 of 65 (23.1%) 1-year old iGBS survivors and in 11 of 207 (5.3%) infants with no history of iGBS. Infants who survived iGBS were 4.23 (95% confidence interval [CI]: 1.65–10.83) times more likely to have neurological sequelae than non-iGBS infants [5, 6].

## AIM AND OBJECTIVES

This article is part of a supplement Every Country, Every Woman, Every Child; Group B Streptococcal Disease Worldwide. The aim is to describe long-term NDI outcomes in iGBS survivors and non-iGBS children aged 5 to 8 years in South Africa, a setting with a high maternal HIV prevalence [3, 5, 6].

## OBJECTIVES

Our objectives are to:

1. Describe iGBS characteristics in early infancy in a cohort of children now aged 5 to 8 years from South Africa.
2. Compare multi-domain and domain specific impairment severity amongst iGBS survivors and children without a history of iGBS, using Griffiths Mental Development Scales-Extended Revised (GMDS-ER) scores.

## METHODS

### Overall Study Design, Setting, and Recruitment Case Definitions

**Figure 1.**
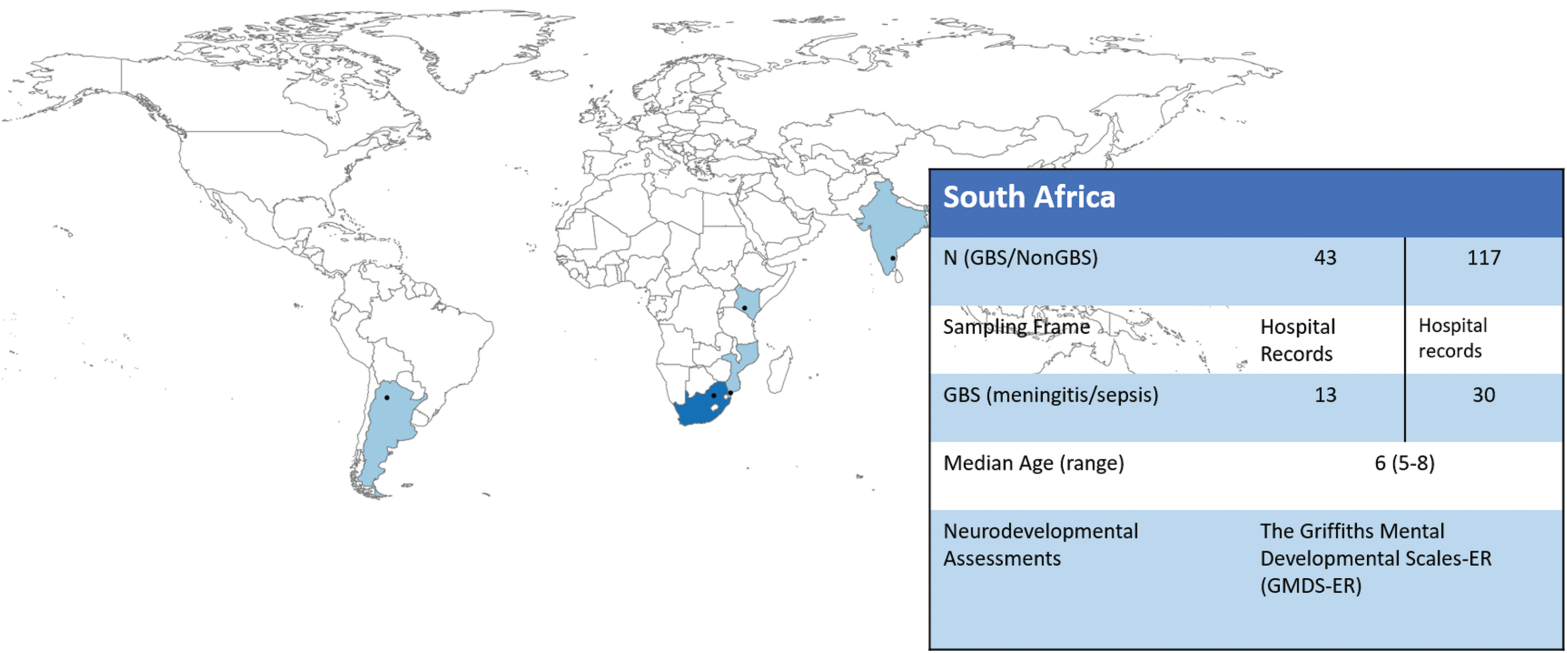
Map of the multi-country iGBS long-term follow-up studies, showing details of the South Africa site. South Africa was one of 5 low and middle-income countries who participated in the study.

We performed a matched cohort study via hospital records (Figure 1), to describe NDI, behavioral and economic outcomes for survivors of iGBS in early infancy, as part of a multi-country study on the long-term outcomes of iGBS survivors in LMIC [7]. The iGBS cohort comprised of children with a history of iGBS (iGBS survivors) and the non-iGBS comparison cohort were children with no known history of iGBS (non-iGBS cohort). The iGBS survivors and non-iGBS children, who were 5 to 8 years old, were identified from participants enrolled for previous studies between 2012 and 2014 [3, 5, 6]. As previously described, iGBS survivors were identified through daily surveillance of the pediatric wards and microbiology services at 3 large academic hospitals in Johannesburg; namely, Chris Hani Baragwanath Academic Hospital, Charlotte Maxeke Johannesburg Academic Hospital and Rahima Moosa Mother and Child Hospital [3, 5, 6]. The non-iGBS cohort were identified through birth registries. In our study setting, the standard-of-antenatal care for the prevention of iGBS in neonates does not include universal screening for recto-vaginal GBS colonization during pregnancy although intrapartum antibiotic prophylaxis (IAP) is provided to women using the risk-based screening approach (such as prematurity, maternal fever, and prolonged rupture of membranes ≥18 hours prior to delivery). The coverage rates with IAP are, however, low in our study setting (~26%) [5].

Non-iGBS children were matched to iGBS survivors for age (±3 months) and sex in a ratio of 3:1 (non-iGBS:iGBS survivors) children. Children with previous GBS meningitis or GBS sepsis (occurring on days 1 to 89 days of age) were recruited for this study. Sepsis was defined as GBS identified on blood culture, while meningitis was defined as: (i) GBS detection by cerebrospinal fluid (CSF) culture or latex agglutination or (ii) a positive blood culture in an infant in whom the CSF cell count was suggestive of meningitis (ie, pleocytosis ≥20 cells/mm^3^ for neonates or ≥10 cells/mm^3^ for infants between 29 and 89 days of age). In the non-iGBS comparison group, non-iGBS infants were selected because they had no clinical signs of possible serious bacterial infection (ie, history of difficulty feeding, history of convulsions, movement only when stimulated, respiratory rate of ≥60 breaths per minute, severe chest in-drawing, temperature ≥37.5°C or ≤35.5°C) and who were not hospitalized for any illness in the first 89 days of age.

The enrollment occurred from October 2019 to January 2021. Multiple attempts by telephone were made by study staff to sequentially contact all the caregivers from the existing participant databases (previous studies undertaken between 2012 and 2014) and schedule an in-person assessment visit for their child at the study facility (Chris Hani Baragwanath Academic Hospital). During the visit, written informed consent from the parent and assent (if appropriate) from the child was obtained.

### Neurodevelopmental Assessments

In our study we used the Griffiths Mental Development Scales-Extended Revised (GMDS-ER) to determine neurodevelopmental outcomes across multiple domains. The GMDS-ER is administered and scored according to a standardized procedure, and it has been validated in South Africa’s multi-cultural environment [8, 9]. The mother or primary caregiver was present during the assessment that was carried out by one of three developmental (GMDS-ER-trained) pediatricians; one pediatrician spoke African languages. An interpreter/ translator was needed for Zulu- and Sesotho-speaking participants to accommodate for language differences when conducting the GMDS-ER assessments. We used the same 3 interpreters/ translators throughout the study. In addition, the interpreters/translators observed assessments done by the developmental pediatrician that spoke African languages to hear how questions were phrased in the African languages. Each participant was assessed once by a single GMDS-ER-trained pediatrician; if needed, consultations between pediatricians occurred to resolve final scores. The testers were blinded to the child’s GBS status.

GMDS-ER has 6 subscales (described in Supplementary Table 1): (A) locomotor, (B) personal-social, (C) language, (D) hand-eye coordination, (E) performance, and (F) practical reasoning. The raw scores of the 6 subscales were converted to the corresponding age equivalent score by using the British norm group. Developmental quotients (DQs) were determined for each subscale by dividing the age equivalent score of the child by their chronological age at the time of testing (DQ = age equivalent score/chronological age × 100). An average of subscale raw scores was used to calculate a global score, the general quotient (GQ). The global GQ was converted to the corresponding age equivalent score by using the British norm group and is referred to as the global DQ. To allow comparisons with other sites using domains, the motor (A, D) and cognitive specific impairment (C, D, E, F) were derived from a combination of the GMDS-ER subscales.

Vision was assessed using the Tumbling E test. For hearing, children initially were screened at 35 dB and 500, 1000, 2000, 4000 Hz with an audiometer; children with abnormal screening results were referred for further testing by a certified audiologist. The Child Behavior Checklist (CBCL) was used to assess emotional-behavioral problems placeholder ref: Chandna iGBSpaper4. Anthropometry was measured and a seizure screening questionnaire was administered. All assessments were conducted by trained assessors. Data were collected on paper forms and then entered into a customized Android table-based app.

Moderate or severe NDI was the primary outcome as a composite of GMDS-ER motor subscales (A, D), GMDS-ER cognition subscales (C, D, E, F), hearing and vision evaluations. Secondary outcomes included mild NDI, any emotional-behavioral problems, and GMDS-ER subscale scores. Impairment severity for each domain is defined in Supplementary Table 2. For a child’s overall NDI, mild impairment is defined as any 2 domains mildly affected, and moderate or severe impairment is defined as any domain moderately or severely affected, or 3 domains mildly affected [10].

Statistical analyses were performed using Stata version 15.2. A normality test was conducted for continuous data. Continuous data with a skewed distribution are described as medians (ranges) and were analyzed using a Kruskal-Wallis test with Dunn’s multiple comparisons post hoc test. Baseline characteristics were compared between the iGBS survivors and the non-iGBS children using χ ^2^/Fisher exact test. NDI risk (95% CI) was quantified for the iGBS survivors and non-iGBS children. Conditional logistic regression was used for the primary analysis to estimate the association of iGBS with moderate or severe NDI controlling for prematurity (37 + weeks vs <37 weeks) and child’s HIV exposure; we did not adjust for age and sex as this was part of the study design. For the analysis related to the association of NDI and clinical syndrome (sepsis vs meningitis), we used a logistical regression adjusting matching variables age and sex, as well as prematurity and child’s HIV exposure, to estimate the association of NDI and clinical syndrome. We controlled for the matching variables in the logistical regression because the distribution may not be the same between sepsis and meningitis. The power for the overall multi-country study was calculated but not for the individual sites; power calculations are described in the published protocol [7]. *P* values of <.05 were considered statistically significant.

## RESULTS

**Figure 2.**
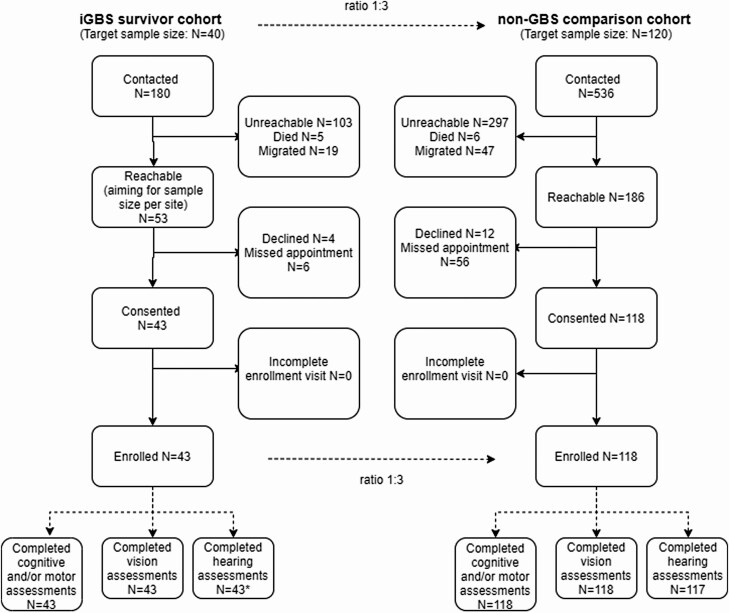
Participant flow of iGBS survivors and non-iGBS children recruited. Out of 180 iGBS survivors contacted, 43 consented for participation and completed the assessment. Out of 536 matched non-iGBS children contacted for participation, 118 children consented and completed neurodevelopmental and vision assessments. One child did not complete the hearing assessment. The final sample size used for the non-GBS comparison group was thus 117.

Of the 180 iGBS survivors, 5 (2.8%) demised after the acute GBS illness, 19 (10.6%) migrated, and 103 (57.2%) were unreachable by telephone. We enrolled 43 iGBS survivors and 117 non-iGBS children (Figures 1 and 2). The GMDS-ER, auditory and visual assessment were completed on 160 children aged between 5 and 8 years (mean: 6.16 years).

Of the 43 iGBS survivors, 13 (30.2%) had meningitis (Table 1). Twenty-one (48.8%) infants presented with late-onset disease. There were no differences in baseline characteristics between iGBS survivors and the non-iGBS comparison cohort with respect to sex (*P* = .989), gestational age (*P* = .356), birth weight (*P* = .107), HIV exposure (*P* = .170), and highest level of education for the main caregiver (*P* = .679, Table 1).

**Table 1.** Description of Baseline Characteristics of Included South African iGBS Survivors and non-iGBS children

	All iGBS Survivors (n = 43)	Non-iGBS (n = 117)	*P*-value
Clinical presentation, n (%)			
Sepsis	30 (69.8)		
Meningitis	13 (30.2)		
GBS onset, n (%)			
Early-onset	22 (51.1)		
Late-onset	21 (48.8)		
Age in years, median (range)	6 (5-8)	6 (5-8)	.204
Sex, n (%)			.989
Female	21 (48.8)	57 (48.7)	
Male	22 (51.2)	60 (51.3)	
Gestational age, n (%)			
≥37	34 (79.1)	100 (85.5)	.356
33–36	4 (9.3)	11 (9.4)	
28–32	5 (11.6)	5 (4.3)	
Don’t know	0 (0.0)	1 (0.8)	
Birthweight, n (%)			
≥2500 g	33 (76.7)	102 (87.2)	.107
<2500 g	10 (23.3)	15 (12.8)	
HIV-exposure status, n (%)			.170
HIV exposed	19 (44.2)	38 (32.5)	
HIV unexposed	24 (55.8)	79 (67.5)	
Highest education for main caregiver, n (%)			
Primary	1 (2.33)	1 (0.85)	.679
Secondary	32 (74.4)	84 (71.8)	
Higher education (university/technical/)	10 (23.3)	32 (27.3)	

Abbreviations: GBS, group B *Streptococcus*; HIV, human immunodeficiency virus; SD, standard deviation.

Six (13.9%) of the iGBS survivors and five (4.3%) of the non-iGBS children had moderate or severe NDI. iGBS exposure was associated with a 5.56 (95% CI: 1.07–28.93; *P* = .041) adjusted odds of moderate or severe NDI at 5–8 years of age compared to the non-iGBS children. Similarly, iGBS survivors (n = 12; 27.9%) were more likely to have any NDI compared to the non-iGBS comparison cohort (n = 5; 4.3%) (adjusted odds ratio [aOR]: 11.51 (95% CI: 2.53–52.42; *P* = .002). A larger proportion of children who had iGBS meningitis (23.1%) had moderate or severe NDI compared to children that had sepsis (10%) during infancy; however, this was not statistically significant (aOR: 3.67; 95% CI: .40–33.40; *P* = .249). Behavioral problems were prevalent in approximately 25% of children and similar between the 2 groups of children (Table 2). Across NDI classifications (none, mild, moderate or severe) for the whole cohort (iGBS and non-iGBS), there were no differences in the language spoken by the children, with Zulu being the most common language spoken (50%) and English the least common (~5%) (*P* = .838).

**Table 2.** Summary of Neurodevelopmental Findings by Domain for iGBS Survivors (n = 43) and Non-iGBS (n = 117) Children Aged 5–8 years in South Africa

	iGBS Sepsis (n = 30)	iGBS Meningitis (n = 13)	All iGBS Survivors (n = 43)	Non-iGBS (n = 117)
Overall NDI^b^ n (%)				
Moderate or Severe	3 (10.0)	3 (23.1)	6 (13.9)	5 (4.3)
Mild	3 (10.0)	3 (23.1)	6 (13.9)	0 (0.0)
Motor n (%)				
Moderate or Severe	0 (0.0)	1 (7.7)	1 (2.3)	0 (0.0)
Mild	5 (16.7)	5 (38.5)	10 (23.3)	6 (5.1)
Cognition n (%)				
Moderate or Severe	3 (10.0)	3 (23.1)	6 (13.9)	4 (3.4)
Mild	6 (20.0)	5 (38.5)	11 (25.9)	26 (22.2)
Vision impairment n (%)				
Moderate or Severe	0 (0.0)	0 (0.0)	0 (0.0)	1 (0.8)
Mild	1 (3.1)	0 (0.0)	1 (2.3)	1 (0.8)
Hearing impairment^a^ n, (%)				
Moderate or Severe	1 (3.1)	1 (9.1)	2 (4.7)	0 (0.0)
Mild	0 (0.0)	0 (0.0)	0 (0.0)	0 (0.0)
Any behavioral problems n (%)	6 (18.7)	5 (45.5)	11 (25.6)	26 (22.2)

Abbreviations: iGBS, invasive group B *Streptococcus*; NDI, neurodevelopmental impairment.

^a^Nine possible hearing impaired assumed to have no impairment.

^b^For overall impairment: Mild impairment is defined as any 2 domains mildly affected. Moderate/severe impairment is defined as any domain moderately or severely affected, or 3 domains mildly affected [10]. The domain and severity definitions are provided in Supplementary Table 2.

**Figure 3.**
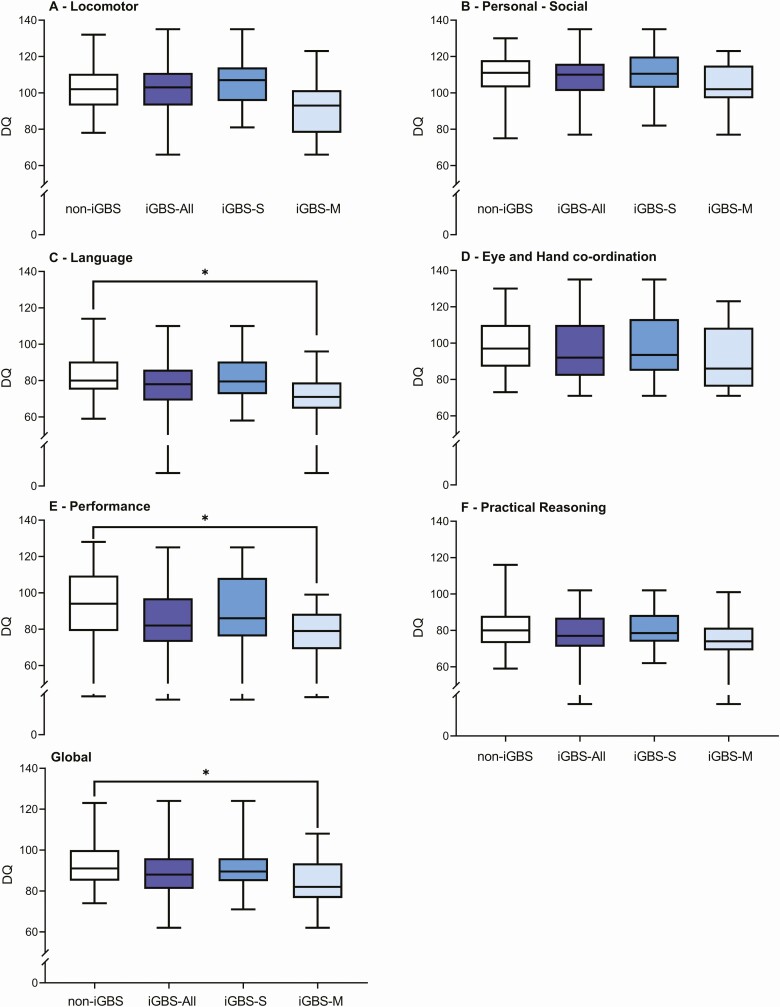
Box and whisker plot between non-iGBS comparison group and all iGBS survivors (meningitis (iGBS-M) and sepsis (iGBS-S)), for Griffiths Mental Developmental Scales-Extended Revised (GMDS-ER) developmental quotient (DQ) scores by subscale A to F. Developmental quotients (DQs) were calculated for each subscale by dividing the age equivalent score per child by their chronological age at the time of testing. DQ = age equivalent score/chronologic age × 100. The global GQ was converted to the corresponding age equivalent score by using the British norm group and is referred to as the global DQ. *P < .05. Two of the iGBS (n = 1 for sepsis and n = 1 for meningitis) survivors had moderate or severe hearing impairment.

The chronological age range for the children was between 5 and 8 years of age. Table 3 shows that iGBS survivors had a higher prevalence of overall (mild, moderate or severe) NDI in the locomotor, eye and hand coordination, performance subscales and global DQ score compared to the non-iGBS cohort (*P* < .05). Figure 3 shows that the median DQ scores for the language (*P* = .034) and performance (*P* = .029) subscales and the global DQ score (*P* = .028) was significantly lower in the iGBS meningitis survivors compared to the non-iGBS cohort. No significant differences were observed for the median DQ subscale scores or median global DQ scores between the iGBS sepsis survivors and the non-iGBS cohort. The raw scores for each subscale and the global GQ are provided in Supplementary Figure 2.

**Table 3.** Griffiths Mental Development Scales Comparing iGBS (n = 43) Survivors and Non-iGBS (n = 117) Children Aged 5–8 years in South Africa

	iGBS Sepsis (n = 30)	iGBS Meningitis (n = 13)	All iGBS Survivors (n = 43)	Non-iGBS (n = 117)	*P*-value^a^
Locomotor					.045
Moderate or Severe (< 70)		1 (7.7)	1 (2.3)		
Mild (70 – 79)		3 (23.1)	3 (6.9)	2 (1.7)	
Personal-social					.176
Moderate or Severe (< 70)					
Mild (70 – 79)		2 (15.4)	2 (4.7)	2 (1.7)	
Hearing and language					.131
Moderate or Severe (< 70)	6 (20.0)	5 (38.5)	11 (25.6)	15 (12.8)	
Mild (70 – 79)	9 (33.3)	6 (46.2)	15 (34.9)	40 (34.2)	
Eye and hand coordination					.001
Moderate or Severe (< 70)	5 (16.7)	4 (30.8)	9 (20.9)	4 (3.4)	
Mild (70 – 79)					
Performance					.032
Moderate or Severe (< 70)	3 (10.0)	3 (23.1)	6 (14.0)	14 (12.0)	
Mild (70 – 79)	9 (33.3)	4 (30.8)	13 (30.2)	17 (14.5)	
Practical reasoning					.336
Moderate or Severe (< 70)	4 (13.3)	3 (23.1)	7 (16.3)	12 (10.3)	
Mild (70 – 79)	12 (40.0)	6 (46.2)	18 (41.9)	46 (39.3)	
Overall DQ					.002
Moderate or Severe (< 70)		2 (15.4)	2 (4.7)		
Mild (70 – 79)	5 (16.7)	2 (15.4)	7 (16.3)	5 (4.3)	

Abbreviations: DQ, developmental quotient; iGBS, invasive group B *Streptococcus*; NDI, neurodevelopmental impairment.

^a^χ ^2^ or Fisher exact test comparing overall NDI in iGBS survivors compared to non-GBS children.

## Discussion

We found that moderate or severe long-term NDI is common in South African children surviving iGBS sepsis or meningitis during infancy, with approximately one tenth of iGBS survivors having moderate or severe NDI at 5–8 years of age, which translates to being 5– 6 times more likely to suffer moderate or severe NDI compared to their peers. Although long-term moderate or severe NDI was especially frequent in survivors of iGBS meningitis, survivors of iGBS sepsis were also found to be affected. The occurrence of NDI in iGBS sepsis survivors is important, because there is a higher incidence of iGBS sepsis than there is of iGBS meningitis. NDI (either mild, moderate or severe) was found in over a quarter (27.9%) of these 5- to 8-year-olds surviving iGBS. Interestingly, we identified similar levels (~20%) of mild cognitive impairment between the iGBS survivors and the non-iGBS children.

Compared to other LMICs included in the multi-country study, the rate of any NDI in our study was similar for iGBS survivors (27.8%) but lower for the non-iGBS children (4.3%). In India, the rate of any NDI impairment was 48.6% in iGBS survivors compared to 38% in non-iGBS children aged 1–14 years, and, in Mozambique, the rate of any NDI impairment in iGBS survivors was 20% compared to 18% in non-iGBS children aged 3–18 years [11, 12]. The studies undertaken in India and Mozambique used different NDI assessments in their cohort of children and thus the NDI rates noted in their cohorts may not be directly comparable to our study undertaken in South Africa. In comparison to recent data from a matched cohort study in a high-income setting (Denmark and Netherlands), we reported a greater percentage of moderate or severe NDI in iGBS survivors, and in particular children with a history of GBS meningitis [13]. There was a high prevalence of NDI in the non-iGBS children in our study, which may be related to factors such as a poor socio-economic environment, HIV exposure, undernutrition, and poor South African formal education infrastructure. In addition, some differences could be attributable to the neurodevelopmental assessment tool used. We used the GMDS-ER tool to assess neurodevelopment and the subscales for Language, Eye and Hand Coordination, Performance and Practical Reasoning in our calculation of cognitive NDI. Previous studies using the GMDS-ER have shown that South African children achieve lower scores on cognitive-orientated subscales compared to British children [8]. The language subscale is considered to be the most intellectual of all the subscales and assesses the growth and development of both receptive and expressive language [14]. Thus, the high prevalence of cognitive NDI detected in the non-iGBS cohort could also be influenced by the GMDS-ER tool being referenced against British children and thus not representative of a poor socio-economic setting, such as in South Africa. Nonetheless, our results indicate that iGBS is a significant and independent cause of NDI.

We previously reported NDI identified in 15 of 65 (23.1%) infants with iGBS at 1 year of age [5, 6]. Our current follow-up assessment performed when the children were 5 to 8 years of age, identified NDI in 27.9% of cases and emphasizes the need for early and regular NDI assessments, so that interventions to reduce the impact can be implemented. One strength of the GMDS-ER is that the various subscales can be scored separately to identify more specific weaknesses. In our study, we noted a difference between iGBS meningitis survivors and non-iGBS children in the language and performance subscales. Using a tool which assessing multiple items, like the GMDS may be useful in focusing interventions.

Although previous studies have shown an increased risk of NDI in HIV-exposed children compared to their HIV-unexposed peers [4], iGBS survivors had an increased odds of NDI even after controlling for HIV exposure. Our study has strengths, notably the multi-country design, linked cohort follow-up of children to 5–8 years and having a counterfactual group with matching of 1 to 3. An important limitation of our study was the large number of children we could not follow-up. Migration from this urban poor township setting may account for some of these cases. We did a risk of bias assessment using the dataset from our previous studies [5, 6], comparing children not included in this study with the children included. We found no significant difference in NDI outcome at one year between these groups (enrolled in current study vs not enrolled in current study), nor any significant difference in maternal age and HIV status, gender, and clinical syndrome. We believe that moderate or severe NDI is unlikely to be more prevalent in the lost to follow-up group because, in our resource-limited setting, caregivers are less likely to return to hospital for assessments if they perceive that their children are well.

## CONCLUSION

Until maternal GBS vaccination or intrapartum antibiotic prophylaxis are universally available, the burden of disease, including NDI after iGBS, remains high worldwide, including in South Africa. Further studies are required to develop simpler, standardized, culturally appropriate, and openly accessible NDI assessments to identify milder impairment during the primary education school years. Implementation research, including economic evaluation, is required to inform scale-up for detection and care of at-risk newborns.

## Supplementary Data

Supplementary materials are available at *Clinical Infectious Diseases* online. Consisting of data provided by the authors to benefit the reader, the posted materials are not copyedited and are the sole responsibility of the authors, so questions or comments should be addressed to the corresponding author.

ciab814_suppl_Supplementary_Materials

## Notes

***Acknowledgements.*** We thank all the study staff for their hard work and dedication to this project. We express appreciation to the LMIC GBS study collaborative group for their input on the research question and overall study design as part of the larger multi-country study:

***Authors’ contributions.*** The GBS study was conceptualized by J. E. L. All site teams contributed to the design of the study protocol and undertook data collection. L.M.H. together with P. P., J .C., J. E. L., B. L., and Z. D. developed the detailed research questions and overall analysis plan for this paper. Analysis was undertaken by L. M. H., P. P., and Z. D. P. P. provided statistical oversight. The manuscript was drafted by L. M. H., S. G. L., and Z. D. All authors reviewed and helped to revise the manuscript. All authors reviewed and agreed the final version.

***GBS LMIC collaborative group for long-term outcomes***. Shabir A. Madhi (Medical Research Council: Vaccines and Infectious Diseases Analytical Unit, Faculty of Health Sciences, University of the Witwatersrand, Johannesburg, South Africa; Department of Science and Technology/National Research Foundation: Vaccine Preventable Diseases, Faculty of Health Sciences, University of the Witwatersrand, Johannesburg, South Africa), Ziyaad Dangor (Medical Research Council: Vaccines and Infectious Diseases Analytical Unit, Faculty of Health Sciences, University of the Witwatersrand, Johannesburg, South Africa), Shannon Leahy (Department of Paediatrics and Child Health, Faculty of Health Sciences, University of the Witwatersrand, Johannesburg, South Africa), Lois Harden (Brain Function Research Group, School of Physiology, Faculty of Health Sciences, University of the Witwatersrand, Johannesburg, South Africa), Azra Ghoor (Department of Paediatrics and Child Health, Faculty of Health Sciences, University of the Witwatersrand, Johannesburg, South Africa), Sibongile Mbatha (Department of Paediatrics and Child Health, Faculty of Health Sciences, University of the Witwatersrand, Johannesburg, South Africa), Sarah Lowick (Department of Paediatrics and Child Health, Faculty of Health Sciences, University of the Witwatersrand, Johannesburg, South Africa), Tamara Jaye (Department of Paediatrics and Child Health, Faculty of Health Sciences, University of the Witwatersrand, Johannesburg, South Africa), Sanjay G Lala (Department of Paediatrics and Child Health, Faculty of Health Sciences, University of the Witwatersrand, Johannesburg, South Africa), Pamela Sithole (Medical Research Council: Vaccines and Infectious Diseases Analytical Unit, Faculty of Health Sciences, University of the Witwatersrand, Johannesburg, South Africa), Jacqueline Msayi (Medical Research Council: Vaccines and Infectious Diseases Analytical Unit, Faculty of Health Sciences, University of the Witwatersrand, Johannesburg, South Africa), Ntombifuthi Kumalo (Medical Research Council: Vaccines and Infectious Diseases Analytical Unit, Faculty of Health Sciences, University of the Witwatersrand, Johannesburg, South Africa), Tshepiso Nompumelelo Msibi (Medical Research Council: Vaccines and Infectious Diseases Analytical Unit, Faculty of Health Sciences, University of the Witwatersrand, Johannesburg, South Africa), Sridhar Santhanam (Department of Neonatology, Christian Medical College, Vellore, India), Hima B. John (Department of Neonatology, Christian Medical College, Vellore, India), Asha Arumugam (Department of Neonatology, Christian Medical College, Vellore, India), Nandhini Murugesan (Department of Neonatology, Christian Medical College, Vellore, India), Nandhini Rajendraprasad (Department of Neonatology, Christian Medical College, Vellore, India), Mohana Priya (Department of Neonatology, Christian Medical College, Vellore, India), Amina Abubakar (Neuroscience Research Group, Department of Clinical Sciences, KEMRI-Wellcome Trust, Kilifi, Kenya; Institute for Human Development, Aga Khan University, Nairobi, Kenya), Carophine Nasambu (Neuroscience Research Group, Department of Clinical Sciences, KEMRI-Wellcome Trust, Kilifi, Kenya), Adam Mabrouk Adan (KEMRI/Wellcome Trust Research Programme, Kilifi, Kenya), Patrick Vidzo Katana (KEMRI/Wellcome Trust Research Programme, Kilifi, Kenya), Eva Mwangome (KEMRI/Wellcome Trust Research Programme, Kilifi, Kenya), Charles R. Newton (Department of Psychiatry, Medical Sciences Division, University of Oxford, Oxford, UK; KEMRI/Wellcome Trust Research Programme), Quique Bassat (Centro de Investigação em Saúde de Manhiça, Maputo, Mozambique; ISGlobal, Hospital Clínic, Universitat de Barcelona, Barcelona, Spain; ICREA, Pg. Lluís Companys 23, 08010 Barcelona, Spain; Pediatrics Department, Hospital Sant Joan de Déu [University of Barcelona], Barcelona, Spain; Consorcio de Investigación Biomédica en Red de Epidemiología y Salud Pública [CIBERESP], Madrid, Spain), Azucena Bardají (Centro de Investigação em Saúde de Manhiça, Maputo, Mozambique; ISGlobal, Hospital Clínic, Universitat de Barcelona, Barcelona, Spain), Justina Bramugy (Centro de Investigação em Saúde de Manhiça, Maputo, Mozambique), Humberto Mucasse (Centro de Investigação em Saúde de Manhiça, Maputo, Mozambique), Celine Aerts (ISGlobal, Hospital Clínic, Universitat de Barcelona, Barcelona, Spain), Sergio Massora (Centro de Investigação em Saúde de Manhiça, Maputo, Mozambique), Romina Libster (Fundación INFANT, Buenos Aires, Argentina), Clara Sánchez Yanotti (Fundación INFANT, Buenos Aires, Argentina), Valeria Medina (Instituto de Maternidad y Ginecología Nuestra Señora de las Mercedes, Argentina), Andrea Rojas (Instituto de Maternidad y Ginecología Nuestra Señora de las Mercedes, Argentina), Daniel Amado (Instituto de Maternidad y Ginecología Nuestra Señora de las Mercedes, Argentina), Conrado J. Llapur (Instituto de Maternidad y Ginecología Nuestra Señora de las Mercedes, Argentina), A. K. M. Tanvir Hossain (Maternal and Child Health Division, International Centre for Diarrhoeal Disease Research, Bangladesh [icddr,b], Dhaka, Bangladesh), Qazi Sadeq-ur Rahman (Maternal and Child Health Division, International Centre for Diarrhoeal Disease Research, Bangladesh [icddr,b], Dhaka, Bangladesh).

Finally, and most importantly we thank the women and children participating in the GBS study and their families, without whom this work would not have been possible.

Ethics and consent to participate. Written informed consent was obtained from parents or legal guardians; assent was also obtained from older children participating in the study. The overarching protocol for this multi-country observational study was granted ethical approval at the London School of Hygiene & Tropical Medicine (approval number 16246), the University of Witwatersrand Human Research Ethics Committee in South Africa (approval number M190241), as well as the institutional review board of the World Health Organization (approval number ERC0.0003169).

Data sharing and transfer agreements were jointly developed and signed by all collaborating partners. The data sets used and/or analyzed during the current study are available from the corresponding author on reasonable request.

***Supplement sponsorship.*** This supplement is sponsored by Bill & Melinda Gates Foundation.

***Financial sources.*** This work was supported by a grant (grant number INV-009018) from the Bill & Melinda Gates Foundation to the London School of Hygiene & Tropical Medicine (PI, Joy Lawn). The funders had no role in study design, data collection and analysis, decision to publish, or preparation of the manuscript.

***Potential conflicts of interest.***. S. A. M. has collaborated on GBS grants funded by GlaxoSmithKline and by Pfizer and received personal fees for being member of its advisory committee; he has also collaborated on a GBS grant funded by Minervax. Z. D., S. G. L., J. M., T. N. M., P. S., N. K., and L. M. H. report subawards from LSHTM to Wits Health Consortium, University of the Witwatersrand, Johannesburg, South Africa. A. G. reports support for attendance of Griffiths III training in November 2019.

All other authors report no potential conflicts. All authors have submitted the ICMJE Form for Disclosure of Potential Conflicts of Interest. Conflicts that the editors consider relevant to the content of the manuscript have been disclosed
