# Supplementary material for: South African Children: A Matched Cohort Study of Neurodevelopmental Impairment in Survivors of Invasive Group B Streptococcus Disease Aged 5 to 8 Years
Source: Clin Infect Dis. 2021 Nov 2;74(Suppl 1):S5–S13. doi: 10.1093/cid/ciab814 (PMC8776309; doi:10.1093/cid/ciab814)
Supplement: ciab814_suppl_Supplementary_Materials [file ciab814_suppl_supplementary_materials.docx]

**SUPPLEMENTARY MATERIAL**

**Supplement Title:** Every Country, Every woman, Every Child; Group B Streptococcal Disease Worldwide

**Paper Title:** South African children: A case cohort study of neurodevelopmental impairment in survivors of invasive Group B Streptococcus disease aged 5- to 8-years-old

**Short Title:** NDI in South African iGBS survivors

Authors: Lois M Harden^1*^, Shannon Leahy ^2^, Sanjay G Lala^2^ , Proma Paul^3,4^, Jaya Chandna^3,4^, Sarah Lowick^2^, Sibongile Mbatha^2^, Tamara Jaye^2^, Barbara Laughton^5^, Azra Ghoor^2^, Pamela Sithole^6^, Jacqueline Msayi^6^, Ntombifuthi Kumalo^6^, Tshepiso Nompumelelo Msibi^6^, Shabir A Madhi^6^, Joy E Lawn^3,4^, Ziyaad Dangor^2,6^

* Corresponding author

Affiliations

1. Brain Function Research Group, School of Physiology, Faculty of Health Sciences, University of the Witwatersrand, Johannesburg, South Africa.

2. Department of Paediatrics and Child Health, Faculty of Health Sciences, University of the Witwatersrand, Johannesburg, South Africa.

3. Maternal, Adolescent, Reproductive & Child Health (MARCH) Centre, London School of Hygiene & Tropical Medicine, London, UK

4. Department of Infectious Disease Epidemiology, London School of Hygiene & Tropical Medicine, London, UK

5. Department of Paediatrics and Child Health, Stellenbosch University, Tygerberg, Western Cape, South Africa

6. South African Medical Research Council: Vaccines and Infectious Diseases Analytics Research Unit, Faculty of Health Sciences, University of the Witwatersrand, Johannesburg, South Africa.

Table of Contents

[Supplementary tables 3](#_Toc81572757)

[Table 1. Description of the Griffiths Mental Development Scales-Extended Revised (GMDS-ER) subscales 3](#_Toc81572758)

[Table 2. Domain and Severity definitions for the South African study 5](#_Toc81572759)

[Supplementary figures 7](#_Toc81572760)

[Supplementary Figure 1 – Box and whisker plot of raw scores and the general quotient (GQ) for Griffiths Mental Developmental Scales-ER (GMDS-ER) by subscale A to F between non-iGBS children and iGBS survivors. 7](#_Toc81572761)

[STROBE Statement—Checklist of items that should be included in reports of cohort studies 8](#_Toc81572762)

[References 10](#_Toc81572763)

# Supplementary tables

### Table 1. Description of the Griffiths Mental Development Scales-Extended Revised (GMDS-ER) subscales

| **Subscale** | **Brief description of activities included in each subscale** |
| --- | --- |
| Subscale A: Locomotor | Assesses a child's gross motor skills, including their ability to balance and coordinate and control movements. Activities include walking up and down stairs, kicking a ball, riding a bike, jumping and skipping (1). |
| Subscale B: Personal -Social | Assesses a child’s proficiency in the activities of daily living, their level of independence and ability to interact with other children (1). Activities include personal cleanliness, competency using cutlery, ability to wash own hands and face, to dress and undress, fasten buttons and knowledge of information, such as date of birth or address (1). |
| Subscale C: Language | Subscale C is known as the hearing and language subscale in the Griffiths Scales, whilst in the GMDS-ER it is simply known as the language subscale, as no hearing items are included (2). Assesses a child’s receptive and expressive language. The items administered include age-appropriate items, such as naming objects and colours, repeating sentences, describing a picture and answering a series of questions about comprehension, similarities and differences (1). Children who perform poorly on this subscale, relative to their own performance on the other subscales, may have speech and/or language deficits or may possibly be suffering from a hearing loss (3). |
| Subscale D: Eye and Hand Co-ordination | Assesses a child’s fine motor skills, manual dexterity and visual monitoring skills. Items of this subscale include threading beads, cutting with scissors, copying shapes and writing letters and numbers (1). |
| Subscale E: Performance | Assesses a child’s visuospatial skills including speed of working and precision. It includes age-appropriate items, such as building bridges and stairs, completion of form boards and pattern making amongst others (1). |
| Subscale F: Practical Reasoning | Assesses a child’s ability to solve practical problems, understanding of basic mathematical concepts and questions about moral and sequential issues (1). Items administered include age-appropriate activities, such as counting and comparison of size, length and height. The scale also assesses a child’s ability to count; their knowledge of the days of the week, their visual sequential skills and understanding of right and wrong (1). |

### Table 2. Domain and Severity definitions for the South African study

| Domain definition | Severity definition | |
| --- | --- | --- |
| Motor | None detected | Composite of GMDS subscales A,D (DQ ≥ 80) |
|  | Mild | Composite of GMDS subscales A,D (DQ 70-79) |
|  | Moderate | Composite of GMDS subscales A,D (DQ 55-69) |
|  | Severe | Composite of GMDS subscales A,D (DQ <55) |
| Cognition | None detected | Composite of GMDS subscales C,D,E,F (DQ ≥ 80) |
|  | Mild | Composite of GMDS subscales C,D,E,F (DQ 70-79) |
|  | Moderate | Composite of GMDS subscales C,D,E,F (DQ 55-69) |
|  | Severe | Composite of GMDS subscales C,D,E,F (DQ <55) |
| Vision | None detected | Visual acuity in best eye 6/6 |
|  | Mild | Visual acuity in best eye <6/12 but better or corresponding visual field loss |
|  | Moderate | Visual acuity in best eye between 6/18 and 6/60, or corresponding visual field loss |
|  | Severe | Visual acuity in best eye between 6/60 and 3/60, or corresponding visual field loss |
|  | Blindness | Visual acuity in best eye <3/60, or corresponding visual field loss |
| Hearing^1^ | None detected | Passed audiometry screen at 35 decibels at 1000, 2000, and 4000Hz **OR** Audiometric hearing threshold level <26 decibel **OR** no or very slight hearing problems; able to hear whispers |
|  | Mild | Audiometric hearing threshold level 26-30 decibel **OR** able to hear and repeat words spoken in normal voice at 1 metre |
|  | Moderate | Audiometric hearing threshold level 31-64.9 decibel **OR** able to hear and repeat words spoken in raised voice at 1 metre |
|  | Severe or deafness | Audiometric hearing threshold level ≥65 decibel **OR** able to hear some words when shouted  **OR** unable to hear or understand even in a shouted voice |
| Behavioural problems | Any | CBCL scores within clinical range ≥1 domain(s) of the problem scales |

^1^ Audiometric ISO values and clinical performance are based on WHO grades of impairment (WHO | Grades of hearing impairment [Internet]. WHO. World Health Organization; [cited 2020 Jun 19]. Available from: <http://www.who.int/deafness/hearing_impairment_grades/en/>)

Griffiths Mental Development Scales-Extended Revised (GMDS-ER); Child Behaviour Checklist (CBCL)

#

# Supplementary figures

### Supplementary Figure 1 – Box and whisker plot of raw scores and the general quotient (GQ) for Griffiths Mental Developmental Scales-ER (GMDS-ER) by subscale A to F between non-iGBS children and iGBS survivors.


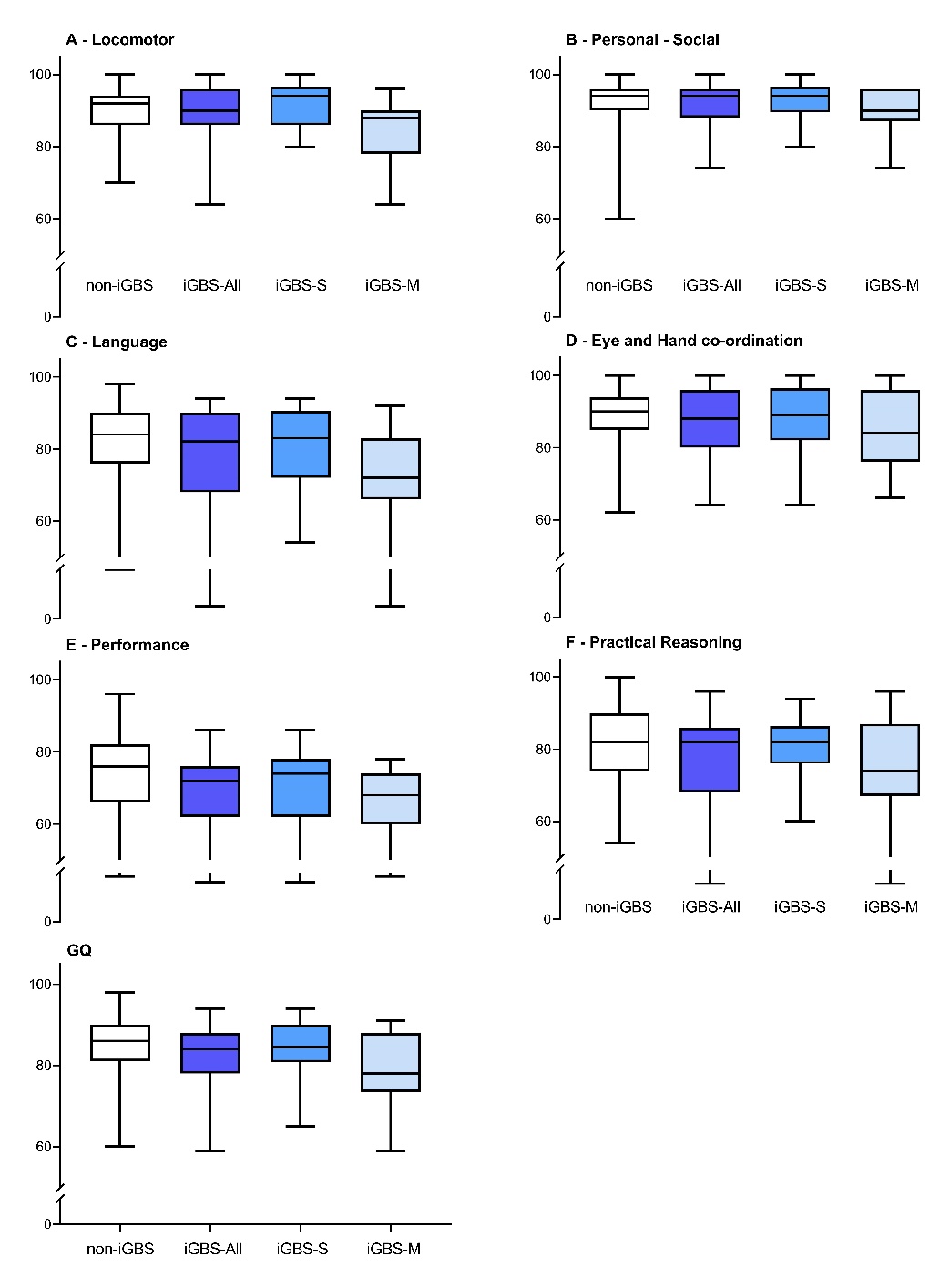


All iGBS survivors, sepsis (iGBS-S), meningitis (iGBS-M). An average of the subscale raw scores was used to calculate a global score, the general quotient (GQ). Two of the iGBS survivors (n=1 sepsis and n=1 meningitis) had moderate to severe hearing impairment.

# STROBE Statement—Checklist of items that should be included in reports of cohort studies

|  | Item No | Recommendation | Page No |
| --- | --- | --- | --- |
| **Title and abstract** | 1 | (*a*) Indicate the study’s design with a commonly used term in the title or the abstract | 1 |
|  |  | (*b*) Provide in the abstract an informative and balanced summary of what was done and what was found | 2 |
| Introduction | | | |
| Background/rationale | 2 | Explain the scientific background and rationale for the investigation being reported | 4 |
| Objectives | 3 | State specific objectives, including any prespecified hypotheses | 4 |
| Methods | | | |
| Study design | 4 | Present key elements of study design early in the paper | 5 |
| Setting | 5 | Describe the setting, locations, and relevant dates, including periods of recruitment, exposure, follow-up, and data collection | 5 |
| Participants | 6 | (*a*) Give the eligibility criteria, and the sources and methods of selection of participants. Describe methods of follow-up | 5,6 |
|  |  | (*b*)For matched studies, give matching criteria and number of exposed and unexposed |  |
| Variables | 7 | Clearly define all outcomes, exposures, predictors, potential confounders, and effect modifiers. Give diagnostic criteria, if applicable | 7 |
| Data sources/ measurement | 8* | For each variable of interest, give sources of data and details of methods of assessment (measurement). Describe comparability of assessment methods if there is more than one group | 6,7 |
| Bias | 9 | Describe any efforts to address potential sources of bias | 6, 10 |
| Study size | 10 | Explain how the study size was arrived at | 7 |
| Quantitative variables | 11 | Explain how quantitative variables were handled in the analyses. If applicable, describe which groupings were chosen and why | 6, 7 |
| Statistical methods | 12 | (*a*) Describe all statistical methods, including those used to control for confounding |  |
|  |  | (*b*) Describe any methods used to examine subgroups and interactions | 7 |
|  |  | (*c*) Explain how missing data were addressed |  |
|  |  | (*d*) If applicable, explain how loss to follow-up was addressed |  |
|  |  | (*e*) Describe any sensitivity analyses |  |
| Results | | |  |
| Participants | 13* | (a) Report numbers of individuals at each stage of study—eg numbers potentially eligible, examined for eligibility, confirmed eligible, included in the study, completing follow-up, and analysed | 7,8 |
|  |  | (b) Give reasons for non-participation at each stage |  |
|  |  | (c) Consider use of a flow diagram |  |
| Descriptive data | 14* | (a) Give characteristics of study participants (eg demographic, clinical, social) and information on exposures and potential confounders | 7,8 |
|  |  | (b) Indicate number of participants with missing data for each variable of interest |  |
|  |  | (c) Summarise follow-up time (eg, average and total amount) |  |
| Outcome data | 15* | Report numbers of outcome events or summary measures over time | 7,8 |

| Main results | 16 | (*a*) Give unadjusted estimates and, if applicable, confounder-adjusted estimates and their precision (eg, 95% confidence interval). Make clear which confounders were adjusted for and why they were included | 7,8 |
| --- | --- | --- | --- |
|  |  | (*b*) Report category boundaries when continuous variables were categorized |  |
|  |  | (*c*) If relevant, consider translating estimates of relative risk into absolute risk for a meaningful time period |  |
| Other analyses | 17 | Report other analyses done—eg analyses of subgroups and interactions, and sensitivity analyses | 7,8 |
| Discussion | | | |
| Key results | 18 | Summarise key results with reference to study objectives | 8.9 |
| Limitations | 19 | Discuss limitations of the study, taking into account sources of potential bias or imprecision. Discuss both direction and magnitude of any potential bias | 10 |
| Interpretation | 20 | Give a cautious overall interpretation of results considering objectives, limitations, multiplicity of analyses, results from similar studies, and other relevant evidence | 9 |
| Generalisability | 21 | Discuss the generalisability (external validity) of the study results | 9 |
| Other information | | | |
| Funding | 22 | Give the source of funding and the role of the funders for the present study and, if applicable, for the original study on which the present article is based | 11, 12 |

*Give information separately for exposed and unexposed group

### References

1. Luiz DM, Barnard A, Knoesen N, *et al*. Griffiths Mental Development Scales – Extended Revised. Administration Manual. Oxford: Hogrefe, ARICD, The Test Agency, 2006.

2. Luiz DM, Barnard A, Knoesen N, *et al*. Griffiths Mental Development Scales – Extended Revised. Administration Manual. Oxford: Hogrefe, ARICD, The Test Agency, 2004

3. Schröder, IA. The performance of hearing impaired children on the Revised Extended Griffiths Scales (Unpublished master’s thesis). University of Port Elizabeth, Port Elizabeth, South Africa, 2004.
